# Mitomycin C treatment induces resistance and enhanced migration via phosphorylated Akt in aggressive lung cancer cells

**DOI:** 10.18632/oncotarget.13237

**Published:** 2016-11-09

**Authors:** Cheng-Ying Shen, Li-Han Chen, Yu-Fen Lin, Liang-Chuan Lai, Eric Y. Chuang, Mong-Hsun Tsai

**Affiliations:** ^1^ Institute of Biotechnology, National Taiwan University, Taipei, Taiwan; ^2^ YongLin Biomedical Engineering Center, National Taiwan University, Taipei, Taiwan; ^3^ Department of Radiation Oncology, University of Texas Southwestern Medical Center, Dallas, Texas, USA; ^4^ Graduate Institute of Physiology, National Taiwan University, Taipei, Taiwan; ^5^ Genome and Systems Biology Degree Program, National Taiwan University, Taipei, Taiwan; ^6^ Bioinformatics and Biostatistics Core, Center of Genomic Medicine, National Taiwan University, Taipei, Taiwan; ^7^ Center for Biotechnology, National Taiwan University, Taipei, Taiwan; ^8^ Institute of Epidemiology and Preventive Medicine, National Taiwan University, Taipei, Taiwan; ^9^ Graduate Institute of Chinese Medical Science, China Medical University, Taichung, Taiwan; ^10^ Graduate Institute of Biomedical Electronics and Bioinformatics, National Taiwan University, Taipei, Taiwan; ^11^ Agricultural Biotechnology Research Center, Academia Sinica, Taipei, Taiwan University, Taipei, Taiwan

**Keywords:** CL1-0, CL1-5, cell migration, mitomycin C, phosphorylated Akt

## Abstract

Since 1984, mitomycin C (MMC) has been applied in the treatment of non-small-cell lung cancer (NSCLC). MMC-based chemotherapeutic regimens are still under consideration owing to the efficacy and low cost as compared with other second-line regimens in patients with advanced NSCLC. Hence, it is important to investigate whether MMC induces potential negative effects in NSCLC. Here, we found that the malignant lung cancer cells, CL1-2 and CL1-5, were more resistant to MMC than were the parental CL1-0 cells and pre-malignant CL1-1 cells. CL1-2 and CL1-5 cells consistently showed lower sub-G1 fractions post MMC treatment. DNA repair-related proteins were not induced more in CL1-5 than in CL1-0 cells, but the levels of endogenous and MMC-induced phosphorylated Akt (p-Akt) were higher in CL1-5 cells. Administering a p-Akt inhibitor reduced the MMC resistance, demonstrating that p-Akt is important in the MMC resistance of CL1-5 cells. Furthermore, we revealed that cell migration was enhanced by MMC but lowered by a p-Akt inhibitor in CL1-5 cells. This study suggests that in CL1-5 cells, the activity of p-Akt, rather than DNA repair mechanisms, may underlie the resistance to MMC and enhance the cells' migration abilities after MMC treatment.

## INTRODUCTION

Lung cancer is the leading cause of cancer-related deaths worldwide [1]. Lung cancer can be categorized into two groups: small-cell lung cancer (SCLC) and NSCLC. NSCLC represents about 85% of lung cancers [2]. The 5-year survival rate of patients with lung cancer is lower than that of patients with other kinds of cancer. For patients with early-stage (I and II) lung cancer, the 5-year survival rate is 54%, while the rate for patients with advanced-stage lung cancer is only 18% [3]. In the past, surgery was the first option for patients with early stage but not advanced-stage NSCLC [4]. Most patients with advanced-stage NSCLC undergo chemotherapy followed by surgery to improve their survival [4].

Mitomycin C (MMC) has been applied in the treatment of NSCLC since 1984 [5]. It has been shown that MMC induces interstrand crosslinks (ICLs) [6], the covalent links between the two strands of DNA, to prevent unwinding of the DNA helix, thus blocking both DNA replication and RNA transcription [7, 8]. Currently, MMC alone is still used as an anti-cancer regimen in cancer patients [9]. However, better survival was demonstrated when MMC was combined with either vinca alkaloid- or platinum-containing drugs in the treatment of patients with advanced-stage NSCLC [10–12]. Moreover, given the efficacy and low cost of MMC and vinorelbine, as compared with other second-line regimens [11], it is likely that MMC-based chemotherapy treatment regimens will be increasingly applied for advanced-stage NSCLC. Unfortunately, even though the initial chemotherapy treatments are successful in advanced-stage NSCLC, the efficacy of MMC-based chemotherapy decreases over time owing to drug resistance in patients with advanced-stage lung cancer [13]. The previous studies showed that DNA repair plays an important role in the development of resistance to chemotherapy drugs in various types of human tumors [14–16]. In addition to DNA repair signalling, the p-Akt plays a key factor in the chemotherapeutic agent-induced resistance in NSCLC [17–19]. Indeed, p-Akt activation is involved in the process of apoptosis inhibition in a variety of human cancer cells [20]. In particular, increased p-Akt expression was associated with stronger chemo-resistance to etoposide, paclitaxel, gemcitabine, cisplatin, trastuzumab [18], and topotecan [21] treatment in NSCLC cell lines. However, neither the importance of DNA repair nor the roles of p-Akt have been explored in advanced lung cancer cells exposed to MMC treatment.

Besides the chemotherapy resistance, metastasis is another major negative characteristic in patients with advanced lung cancer after chemotherapy. One recent study indicated that MMC retarded the migration abilities of human corneal fibroblasts [22] but no study has reported the effects of MMC on lung cancer cells with high metastasis abilities. Thus, four lung adenocarcinoma sublines, namely CL1-0, CL1-1, CL1-2, and CL1-5, which exhibit progressively invasive properties via an *in vitro* selection process [23, 24], are suitable cell models for studying the effects of MMC in lung cancer cells. Among these CL cell lines, CL1-5 cells are the most invasive [23, 25] and express higher endogenous expression levels of p-Akt than CL1-0 cells [26]. Moreover, the p-Akt induced via the overexpression of T-LAK Cell-Originated Protein Kinase (TOPK) accompanied by the increased invasion of CL1-0 cells [27] indicated that the activated p-Akt might enhance the cells' migration abilities.

In order to better understand the potential negative effects induced by MMC in NSCLC, in the present study, we performed clonogenic, apoptosis, and cell-cycle distribution assays on CL1-0, CL1-1, CL1-2, and CL1-5 cells. Then, we examined several proteins involved in different kinds of DNA repair signalling to determine whether DNA repair mechanisms participate in MMC resistance. Finally, we applied a p-Akt inhibitor to examine the importance of activated p-Akt in the cell migration and cell proliferation processes of CL1-0 and CL1-5 cells with or without MMC treatment. Through our study, we hope to identify new methods of improving the efficacy of chemotherapy treatments for aggressive cancer.

## RESULTS

### CL1-2 and CL1-5 cells were more resistant to MMC than were CL1-0 and CL1-1 cells

**Figure 1:**
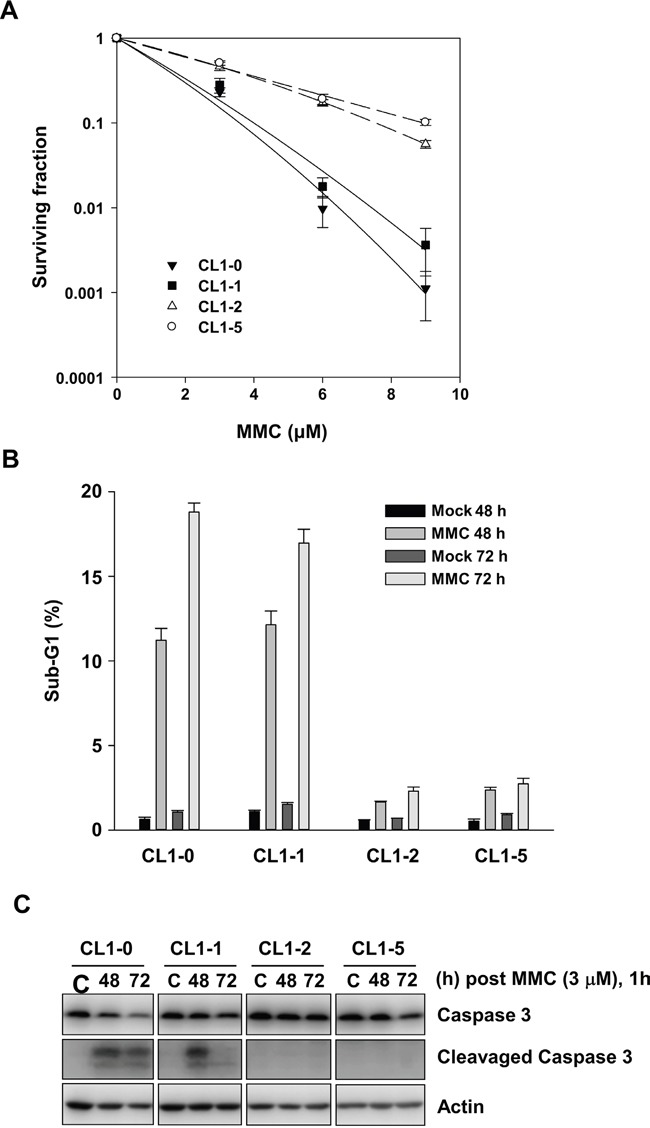
The effects of MMC on CL1-0, CL1-1, CL1-2, and CL1-5 cells **A.** Colony numbers of CL1-0, CL1-1, CL1-2, and CL1-5 cells at the indicated concentration of MMC treatment (n = 3). **B.** The percent of sub-G1 was measured by propidium iodide staining and flow cytometry following 1 h treatment with 3 μM MMC in CL1-0, CL1-1, CL1-2, and CL1-5 cells (n = 3). **C.** Immunoblot analysis of full-length and cleaved caspase 3 products in CL1-0, CL1-1, CL1-2, and CL1-5 cells at 48 or 72 h after 3 μM MMC treatment. C: control, cells without MMC treatment. Loading control: actin.

To examine the MMC-induced cytotoxicity, we performed clonogenic assays to analyse the CL1-0, CL1-1, CL1-2, and CL1-5 cells after exposure to different concentrations of MMC. The results showed that parental CL1-0 and pre-malignant CL1-1 cells were 10-fold more sensitive to treatment with 9 μM MMC than were the derivative CL1-2 and CL1-5 cells (Figure 1A). This indicated that CL1-2 and CL1-5 cells were more resistant to MMC than were CL1-0 and CL1-1 cells.

Next, we analysed the sub-G1 fraction for apoptotic cells. After treatment with 3 μM MMC, the percentage of sub-G1 fraction was significantly more increased in CL1-0 and CL1-1 cells than it was in CL1-2 and CL1-5 cells at 48 or 72 h (Figure 1B). In accordance with the results of the sub-G1 assay, cleaved caspase 3 was detected in CL1-0 and CL1-1 cells but not in CL1-2 and CL1-5 cells at 48 and 72 h after 3 μM MMC treatment (Figure 1C). The results showed that more of the CL1-0 and CL1-1 cells became apoptotic by MMC treatment than did the CL1-2 and CL1-5 cells.

### MMC induced longer G2/M arrest in CL1-0 cells

**Figure 2:**
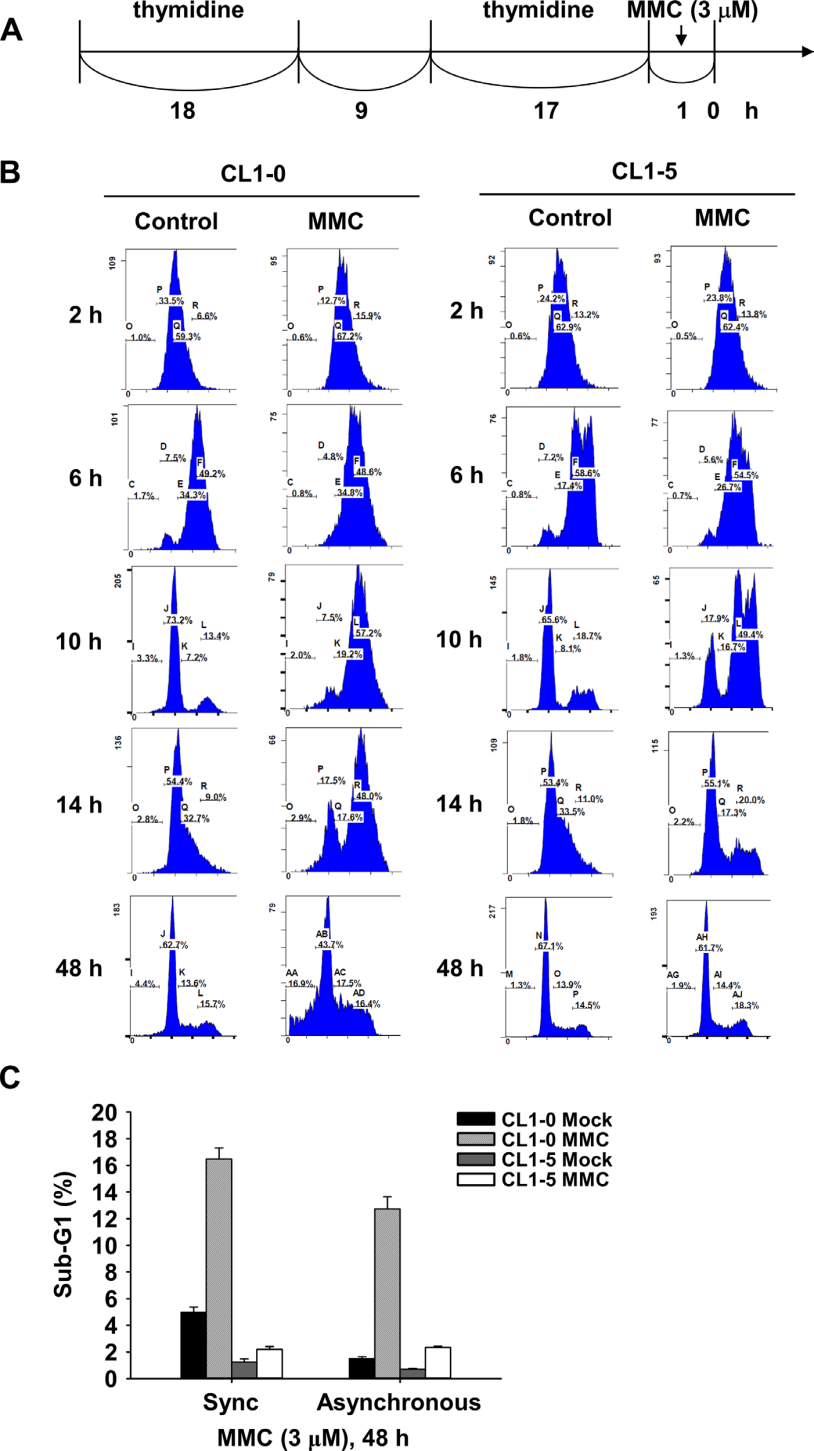
Cell cycle distribution of synchronized CL1-0 and CL1-5 cells post 3 μM MMC treatment **A.** Time scale of double thymidine block and MMC treatment. **B.** Cell cycle distribution of CL1-0 and CL1-5 cells at the indicated time points post MMC (3 μM) treatment in synchronized cells. **C.** Sub-G1 analysis following 1 h treatment with 3 μM MMC in synchronous or asynchronous CL1-0 and CL1-5 cells (n = 3).

We selected CL1-0 and CL1-5 cells to further examine the cell cycle distribution after MMC treatment. First, we synchronized CL1-0 and CL1-5 cells using double thymidine treatment (Figure 2A), and then we performed a flow cytometry analysis to determine the cell cycle distribution. After CL1-0 and CL1-5 cells were released from double thymidine synchronization followed by MMC treatment for 1 h, both cell lines were in the S phase after 2 h of MMC treatment (Figure 2B). Then, we induced G2/M arrest in both CL1-0 and CL1-5 cells after 6 to 10 h of MMC treatment. However, more CL1-0 cells, but not CL1-5 cells, were still arrested in the G2/M phase at 14 h after MMC treatment (Figure 2B). Because longer G2/M arrest durations were observed in CL1-0 cells after MMC treatment, we further analysed the level of phosphorylated Chk2 (p-Chk2) in CL1-0 and CL1-5 cells. The results showed that CL1-0 had higher p-Chk2 (T38) activity than did CL1-5 cells at the indicated time points after MMC treatment (Figure S1). Moreover, the percentage of apoptotic cells in the synchronous cells was similar to the percentage in asynchronous cells with or without MMC treatment in CL1-0 and CL1-5 cells at 48 h (Figure 2C). Therefore, we performed the remaining experiments without double thymidine blocking.

### MMC induced Fanconi anaemia and non-homologous end-joining repair signalling in CL1-0 but not CL1-5 cells

**Figure 3:**
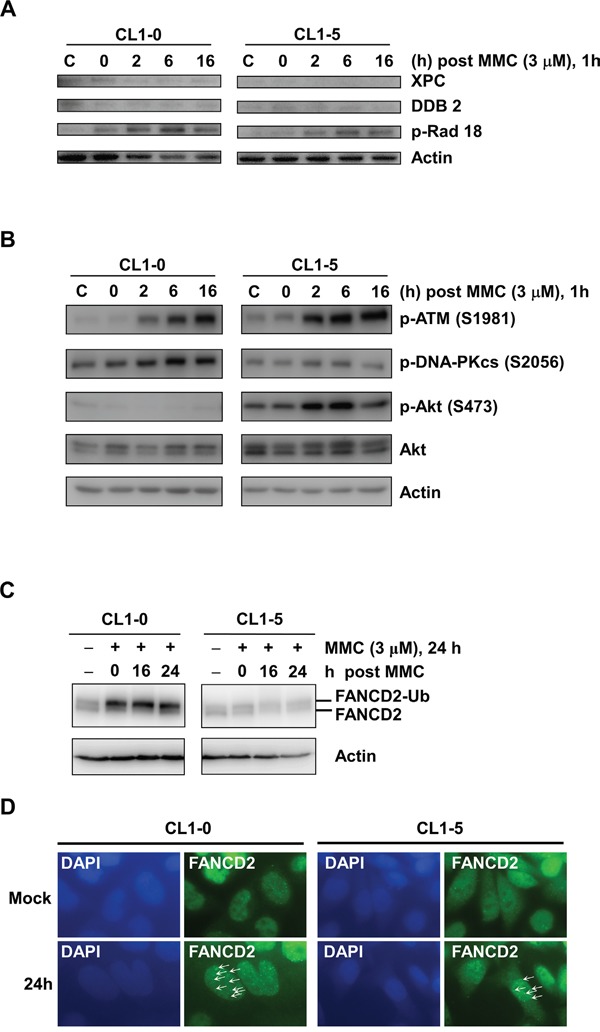
Western blot analysis of DNA damage responses in Cl1-0 and CL1-5 cells post 3 μM MMC treatment **A.** Immunoblot analysis of XPC, DDB2, and p-Rad18 (S403) in CL1-0 and CL1-5 cells at the indicated time points post 3 μM MMC treatment. C: control, cells without MMC treatment. Loading control: actin. **B.** Immunoblot analysis of p-ATM (S1981), p-DNA-PKcs (S2056), p-Akt (S473), and Akt in CL1-0 and CL1-5 cells at different time points post 3 μM MMC treatment. C: control, cells without MMC treatment. Loading control: actin. **C.** Immunoblot analysis of FANCD2 in CL1-0 and CL1-5 cells at the indicated time points post 3 μM MMC treatment.FANCD2-Ub: ubiquitinated FANCD2. Loading control: actin. **D.** Representative images of CL1-0 or CL1-5 cells with or without 3 μM MMC treatment for FANCD2 nuclear foci. Arrows: FANCD2 foci. Green: anti-FANCD2 antibody. Blue: DAPI.

Previous studies indicated that MMC induced DNA interstrand crosslinks to activate several repair pathways such as the nucleotide excision repair (NER), homologous recombination (HR), translesion synthesis (TLS), and Fanconi anaemia (FA) repair pathways [7, 28]. Differences in DNA repair activity may result in the observed differential MMC-induced cytotoxicity between CL1-0 and CL1-5 cells. Moreover, based on our results showing a longer G2/M arrest in CL1-0 than in CL1-5 cells at 14 h, we further studied the activities of DNA repair enzymes within 16 h after MMC treatment. The results of immunoblot analyses showed that xeroderma pigmentosum, complementation group C (XPC), and damage-specific DNA binding protein 2 (DDB2) in the NER pathway could not be induced after MMC treatment (Figure 3A). In addition, p-Rad18 (S403) for TLS repair signalling (Figure 3A), as well as p-ATM (S1981) for HR repair signalling (Figure 3B), were activated in both CL1-0 and CL1-5 cells at the indicated time points after treatment with 3 μM MMC (Figure 3A). Interestingly, the p-DNA-PKcs at S2056 for non-homologous end joining (Figure 3B) and the ubiquitination of Fanconi anaemia complementation group D2 (FANCD2) for FA repair (Figure 3C) showed higher endogenous expression levels and were activated in CL1-0 cells but not in CL1-5 cells after MMC treatment. We also observed similar expression profiles of FANCD2 and p-ATM (S1981) in CL1-1 and CL1-2 cells (Figure S2). As FANCD2 can bind with other FA proteins to form a complex for repairing DNA interstrand crosslinks [29], we examined the level of FA repair by the number of FANCD2 foci in the nuclei. We found that more FANCD2 nuclear foci were detected in CL1-0 cells than in CL1-5 cells at 24 h after MMC treatment (Figure 3D). This indicated that more FA repair were activated in CL1-0 cells than in CL1-5 cells at 24 h after MMC treatment.

### Activation of p-Akt in CL1-5 cells reduced the MMC-induced cytotoxicity

**Figure 4:**
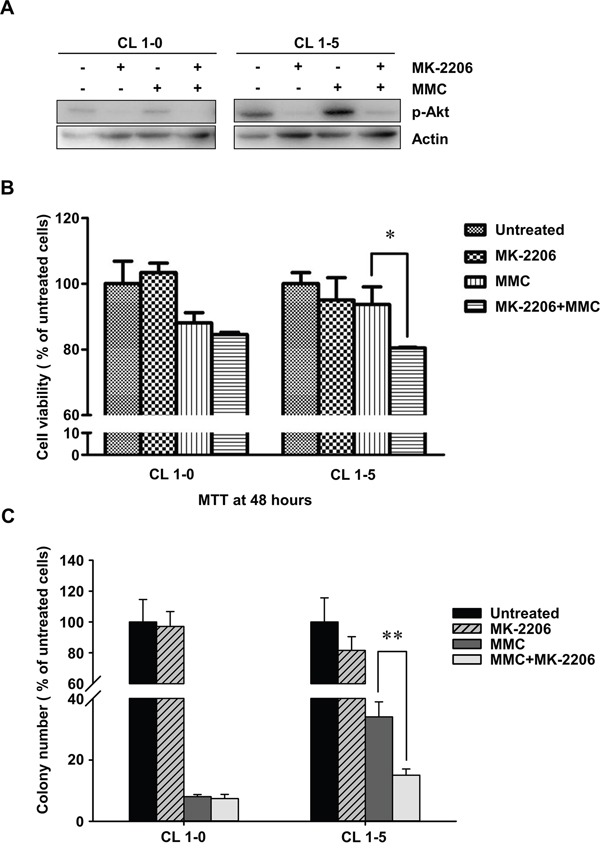
The cell proliferation rates of CL1-0 and CL1-5 cells without p-Akt after MMC treatment **A.** The activity of p-Akt was measured by immunoblot analyses in MK-2206-treated or MK-2206-untreated CL1-0 and CL1-5 cells with or without MMC treatment. Loading control: actin. **B.** The cell proliferation rates of CL1-0 and CL1-5 cells that were untreated, treated with MK-2206, treated with MMC, or treated with a combination of MK-2206 and MMC. *P < 0.05, n = 3. **C.** Colony formation of CL1-0 and CL1-5 cells that were untreated, treated with MK-2206, treated with MMC or with a combination of MK-2206 and MMC. **P < 0.01, n = 4. All values are represented as mean +/− SEM.

Many studies have indicated that constitutive activation of Akt promotes cell survival and resistance to chemotherapeutic agents [30, 31]. Hence, we examined the activity of p-Akt in CL1-5 cells with or without MMC treatment. The results showed that the expression levels of total Akt were similar between CL1-0 and CL1-5 cells with or without MMC treatment. However, CL1-5 cells had both a higher level of endogenous p-Akt (S473) and strong activation of p-Akt after MMC treatment compared to CL1-0 cells (Figure 3B). Therefore, we hypothesized that the activation of p-Akt in CL1-5 cells after MMC treatment might play a crucial role against MMC-induced cytotoxicity. To test this hypothesis, we treated cells with a p-Akt inhibitor, MK-2206, which reportedly represses p-Akt levels [32]. The results showed that p-Akt was reduced in MK-2206-treated CL1-0 and CL1-5 cells with or without MMC treatment (Figure 4A). The MK-2206 treatment did not affect cell viability in either the CL1-0 or CL1-5 cells without MMC (Figure 4B). Moreover, the cell viability of MMC-treated CL1-0 cells was not significantly different between cells that were or were not treated with MK-2206 (Figure 4B). In contrast, MK-2206 significantly decreased the cell viability in MMC-treated CL1-5 cells (*P* < 0.05) (Figure 4B). Likewise, MK-2206 significantly reduced the number of colonies in MMC-treated CL1-5 cells (*P* < 0.01) but not in MMC-treated CL1-0 cells (Figure 4C). These results indicated that p-Akt is important for reducing the cytotoxicity induced by MMC in CL1-5 cells.

### MMC increased the migration abilities of CL1-5 but not CL1-0 cells

**Figure 5:**
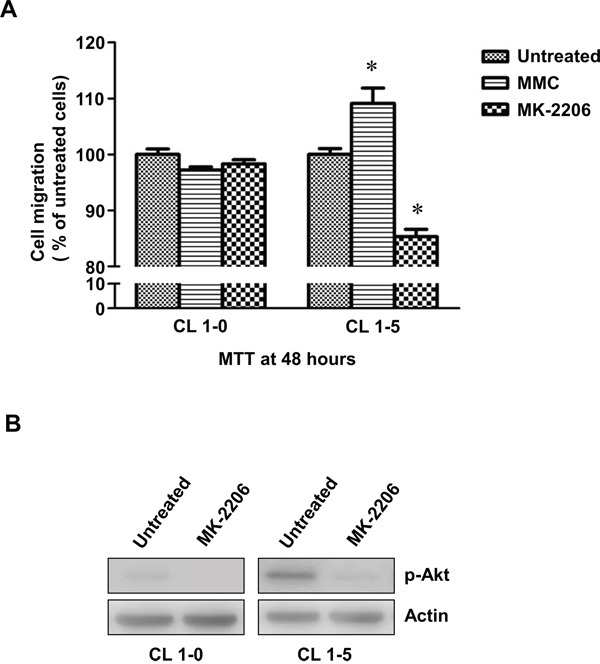
The migration of CL1-0 and CL1-5 cells without p-Akt after MMC treatment **A.** The cell migration of CL1-0 and CL1-5 cells that were untreated, treated with MMC, or treated with MK-2206. *P < 0.05, n = 3. **B.** Immunoblot analysis for confirming the decrease in p-Akt by MK-2206 treatment in CL1-0 and CL1-5 cells. Loading control: actin. All values are represented as mean +/− SEM.

CL1-5 cells are more aggressive with higher migration abilities than CL1-0 cells. Therefore, we further analysed the migration abilities of CL1-0 and CL1-5 cells with or without MMC treatment. The results of transwell assays showed that fewer CL1-0 cells migrated through the permeable membrane after MMC treatment (*P* < 0.05) (Figure 5A). In contrast, MMC treatment was able to induce migration in CL1-5 cells (Figure 5A). More importantly, after the level of p-Akt was reduced by MK-2206 in CL1-0 and CL1-5 cells (Figure 5B), the migration abilities of CL1-5 cells but not CL1-0 cells were dramatically decreased (Figure 5A). These results indicated that MMC could promote migration in the more aggressive CL1-5 cells and that p-Akt may be critical in regulating MMC-induced cell migration.

## DISCUSSION

Chemotherapeutic resistance and metastasis are notable issues in cancer therapy. Furthermore, the previous studies indicated that anticancer- and chemopreventive-agents could induce carcinogenesis or tumor progression [33–35]. MMC, a well-known anticancer drug, was also known as a carcinogen in animal models [36, 37]. The current study provides the first evidence that MMC induces p-Akt, which in turn induces resistance to MMC and enhances the migration abilities of aggressive CL1-5 cancer cells. The efficiency of DNA repair and the activation of survival signalling are two major mechanisms that correlate the efficacy of chemotherapeutic agents. However, the DNA repair mechanisms that were induced by MMC in CL1-5 cells may not be the main mechanisms underlying MMC resistance. For instance, we found that the endogenous levels of MMC-induced p-Akt were higher in CL1-5 cells than they were in CL1-0 cells, and we only detected decreased cell viability in CL1-5 cells following p-Akt inhibition. These results indicate that survival signalling, that is p-Akt but not DNA repair signalling, plays a critical role in MMC resistance in CL1-5 cells. Moreover, we observed that MMC enhanced cell migration in CL1-5 cells. Since p-Akt increased migration in other types of cells [38–40], it was not surprising that MMC increased the migration abilities of CL1-5 cells via the activation of p-Akt, while the addition of a p-Akt inhibitor reduced these abilities.

Although p-Akt is a key factor involved in the resistance to chemotherapeutic agents including cisplatin [17, 41], doxorubicin [41], mitoxantrone [41], paclitaxel [41], etoposide [18], and 5-fluorouracil [41] in NSCLC, the effects of p-Akt on MMC resistance had not been reported before the current study. Kandel et al. revealed that the activities of p-Akt overcame DNA damage induced G2/M arrest to permit survival and proliferation of cells with accumulated DNA mutations in the surviving cell population [42]. Similarly, CL1-5 not only exhibited the higher endogenous and MMC-induced p-Akt levels but passed G2/M arrest faster than did CL1-0 cells after MMC treatment. Accordingly, the enriched p-Akt was important to allow CL1-5 cells to escape the MMC induced G2/M arrest and may proceed through the cell cycle rapidly without DNA repair mechanisms activation. Therefore, the fast replicated CL1-5 cells may contain high MMC tolerance with accumulated DNA mutations which cannot be detected by FANCD2 foci assay at 24 h after MMC treatment. In addition, p-Akt could promote cell survival by direct phosphorylation of BAD [43], which dissociated BAD from the Bcl-2/Bcl-X complex and lose the BAD-related apoptotic function [44]. Moreover, p-Akt could also trigger survival signalings via activation of NF-κB related survival genes [45]. Thus, the MMC-induced high level of p-Akt might either inhibit pro-apoptotic proteins or activate pro-survival genes to reduce MMC induced cytotoxicity in CL1-5 cells. Indeed, our results indicate that using p-Akt inhibitors could improve the efficacy of MMC in lung cancer cells with high endogenous p-Akt expression.

Metastasis is an aggressive feature of cancer [46, 47] and it causes 90% of cancer-related deaths in clinical cases. The previous study also indicated that anticancer drugs treatment can increase further oncogenic characteristics, such as metastasis and invasiveness [48]. Although cisplatin and paclitaxel were linked to metastatic effects via the upregulation of octamer-binding protein 4, vascular endothelial growth factor receptor 1, and matrix metalloproteinase 9 [49–51], the effects of MMC on migration in cancer cells had not been reported previously. The phosphoinositide 3-kinase/Akt pathway regulates both the survival and movement of cells [52, 53]. Moreover, the level of p-Akt was found to be much higher in metastatic breast cancer cells than in non-metastatic tumor cells [54]. Therefore, it was reasonable that MMC increased both the level of p-Akt and migration and that the p-Akt inhibitor conversely decreased migration in CL1-5 cells in our study. Our results suggest that MMC-induced p-Akt plays an important role in promoting the migration of CL1-5 cells. We also believe that additional migration signalling pathways may participate in the MMC-induced mechanisms in CL1-0 and CL1-5 cells; however, this requires further study.

To repair chemotherapeutic agent-induced DNA damage, cancer cells can activate the NER, HR, TLS, and FA repair pathways, which cooperate with each other [55]. NER may be defective in CL1-0 and CL1-5 cells owing to the unchanged levels of NER-related proteins, including XPC and DDB2, following MMC treatment. This is not surprising because genetic mutations, variations, or silencing of NER pathway-related genes can increase the cancer risk by affecting repair efficacy [56, 57]. Besides NER, several proteins involved in the HR and TLS signalling pathways were activated in both CL1-0 and CL1-5 cells after MMC treatment. Moreover, MMC induced fewer FA and non-homologous end-joining DNA repair pathway proteins in CL1-5 cells than it did in CL1-0 cells. Since CL1-5 cells did not induce more DNA repair enzyme activity than CL1-0 cells after MMC treatment, the higher resistance of CL1-5 cells to MMC was likely not related to the DNA repair ability. These results suggest that the activated DNA repair mechanisms may not be as important as the expression of p-Akt in the development of resistance to MMC treatment in CL1-5 cells. However, additional studies are needed, because we only examined a few of the DNA repair pathway members in the present study.

In sum, the present study revealed that the level of p-Akt is an important factor underlying MMC resistance and that MMC seemed to enhance the migration abilities of aggressive CL1-5 cancer cells through the induction of p-Akt. Since cancer metastasis is a negative factor for cancer therapy, treating aggressive cancer with p-Akt-inducing chemotherapeutic agents may need more consideration. Moreover, several studies provided feasibilities for combination of p-Akt inhibitor with other anticancer drugs, such as Erlotinib or Gefitinib, as a viable therapeutic strategy in treating NSCLC [58–60]. Therefore, the present study suggests that the efficacy of chemotherapy may be improved by combining MMC with a p-Akt inhibitor when treating aggressive lung cancer.

## MATERIALS AND METHODS

### Cell culture

Human lung adenocarcinoma cell lines, namely CL1-0, CL1-1, CL1-2, and CL1-5, were cultured in RPMI-1640 medium (ThermoFisher, MA, USA) with 10% foetal bovine serum (FBS, ThermoFisher) and penicillin and streptomycin (100 mg/mL each, ThermoFisher) at 37°C in a humidified atmosphere of 5% CO2.

### MMC treatment and clonogenic assay

Exponentially growing cells were treated with MMC at the indicated concentrations for 1 h at 37°C. After treatment, variant cell numbers were seeded and cultured for 10 days to yield approximately 30–60 colonies per 60-mm dish (Corning, MO, USA). Colonies were then fixed, stained, and counted. A colony was considered to contain at least 50 cells.

### Double thymidine block and flow cytometry analysis

About 25–30% confluent cells were cultured in normal medium with 2 mM thymidine for 18 h, then washed with phosphate-buffered saline (PBS) twice, and re-cultured in normal medium for 9 h followed by an additional incubation with 2 mM thymidine for 17 h. CL1-0 cells and CL1-5 cells were synchronized in G1/S phases with 76–79% of the population by this point. After the cells were released from the thymidine block, they were harvested at the indicated time points and fixed in 70% ethanol overnight. Fixed cells were washed with PBS and re-suspended in propidium iodide (PI) solution (0.1 mg/mL RNase A, 0.1% Triton X-100, 20 μg/mL PI in PBS). After incubation in the PI solution for 30 min at room temperature, the DNA content was measured by a FACScan flow cytometer (BD, MA, USA).

### Immunoblotting

Cell pellets were re-suspended and incubated in lysis buffer (50 mM Tris 7.5, 0.4 M NaCl, 1% Tween 20, 1% NP40, 50 mM sodium orthovanadate, 0.25 M β–glycerophosphate, 0.5 mM dithiothreitol and protease inhibitors) for 30 min and then sonicated at 4°C. The total protein amount was measured by the Bradford method with a protein assay kit (Bio-Rad, CA, USA). Equal amounts of protein from the lysate were loaded and separated by 10% sodium dodecyl sulphate polyacrylamide gel electrophoresis and transferred onto nitrocellulose membranes (Millipore, CA, USA). The blots were hybridized with primary antibodies against caspase 3 (Millipore), β-actin (Cell Signaling Technology, MA, USA), XPC (Cell Signaling Technology), DDB2 (Cell Signaling Technology), p-Rad18 (Cell Signaling Technology), p-ATM (S1981) (Cell Signaling Technology), p-Chk2 (T68) (Cell Signaling Technology), p-DNA-PKcs (S2056) (Cell Signaling Technology), p-Akt (S473) (Cell Signaling Technology), and Akt (Cell Signaling Technology), respectively. After incubation with the horseradish peroxidase-conjugated secondary antibodies (Cell Signaling Technology), the horseradish peroxidase activity was detected by an enhanced chemiluminescence system (UVP BioSpectrum Imaging System).

### Immunofluorescence staining

Cells were fixed with 4% paraformaldehyde in PBS for 15 min, permeabilized with 0.3% Triton X-100 in PBS for 10 min, and blocked with 5% normal goat serum in PBS for 1 h at room temperature. The cells were incubated with primary FANCD2 antibody (Cell Signaling Technology) for 1 h, washed three times with PBS, and then incubated with Alexa Fluor 488-Anti- and Texas Red-conjugated secondary antibodies for 1 h (Invitrogen). Then, cells were washed three times with PBS and mounted in Vectashield mounting medium with 4′,6-diamidino-2-phenylindole (Vector Laboratories).

### MTT assay

We seeded 2 × 104 cells on 24-well plates and harvested the cells daily to measure the cell proliferation. Next, 100 μL of 5 mg/mL (3-(4,5-Dimethylthiazol-2-yl)-2,5-diphenyltetrazolium bromide [MTT]; SIGMA) were added to the cells, and the cells were incubated at 37°C for 3.5 h. Then, the medium was carefully removed without disturbing the cells. The formed insoluble formazan was dissolved with 400 μL of MTT solvent (4 mM HCl mixed with isopropanol), and shaken at room temperature for 15 min. Absorbance was read at 590 nm by VICTOR multi label plate reader (PerkinElmer, MA, USA).

### Transwell assay

Cells were starved with serum-free medium for 24 h. The 6.5-mm-diameter cell culture inserts (8 μm pore size, Corning.) were used to perform transwell assays in 24-well plates. We seeded 1 × 10^5^ cells in the upper chamber of the filter in 200 μL of FBS-free medium. In the lower chamber, we added 0.75 mL of complete medium containing 10% FBS. After 16 h, cells were fixed for 15 min at room temperature with 10% acetic acid and 10% methanol, and washed with 1× PBS. The non-migrating cells on the inner transwell membrane were wiped carefully using a cotton swab and stained with 1 mL of 0.5% crystal violet for 30 min, and then washed by water. The stained cells were solubilized with 10% acetic acid and quantitated on a microplate reader at 580 nm.

### Statistical analysis

Two-tailed Student's *t*-tests were applied to all of the data in this study. Differences were considered to be significant if *P* < 0.05. All values in the text and figures are presented as the mean ± the standard deviation. The sample sizes that we chose are described in the figure legends.

## SUPPLEMENTARY FIGURES


