# Supplementary material for: Mitomycin C treatment induces resistance and enhanced migration via phosphorylated Akt in aggressive lung cancer cells
Source: Oncotarget. 2016 Nov 9;7(48):79995–80007. doi: 10.18632/oncotarget.13237 (PMC5346766; doi:10.18632/oncotarget.13237)
Supplement: Supplementary file 1 [file oncotarget-07-79995-s001.pdf]

Mitomycin C treatment induces resistance and enhanced migration via phosphorylated Akt in aggressive lung cancer cells

SUPPLEMENTARY FIGURES

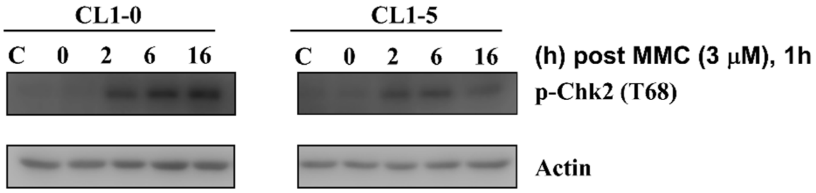

Supplementary Figure S1: Immunoblot analysis of p-Chk2 (T68) in CL1-0 and CL1-5 cells at the indicated time points post 3 μM MMC treatment. C: control, cells without MMC treatment. Loading control: actin.

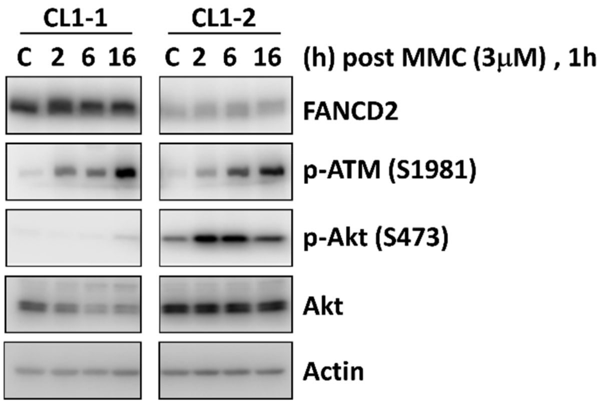

Supplementary Figure S2: Immunoblot analysis of FANCD2, p-ATM (S1981), p-Akt (S473), and Akt in CL1-1 and CL1-2 cells at the indicated time points post 3 µM MMC treatment. Loading control: actin.
